# *N*-Acetyl-*N*-[2,4-dicyano-1-(4-meth­oxy­phen­yl)-9,10-dihydro­phenanthren-3-yl]acetamide

**DOI:** 10.1107/S160053681201210X

**Published:** 2012-03-24

**Authors:** Abdullah M. Asiri, Hassan M. Faidallah, Shaeel A. Al-Thabaiti, Seik Weng Ng, Edward R. T. Tiekink

**Affiliations:** a Chemistry Department, Faculty of Science, King Abdulaziz University, PO Box 80203, Jeddah, Saudi Arabia; b The Center of Excellence for Advanced Materials Research, King Abdulaziz University, Jeddah, PO Box 80203, Saudi Arabia; c Department of Chemistry, University of Malaya, 50603 Kuala Lumpur, Malaysia

## Abstract

In the title compound, C_27_H_21_N_3_O_3_, the cyclo­hexa-1,3-diene ring has a screw-boat conformation, and the fused ring system is folded, the dihedral angle between the outer benzene rings being 27.61 (6)°. The *N*-acetyl­acetamide residue (r.m.s. deviation = 0.0935 Å) has an *anti* conformation and is essentially perpendicular to the benzene ring to which it is connected [dihedral angle = 89.14 (6)°]; the meth­oxy­benzene group is also twisted out of this ring [dihedral angle = 59.47 (7)°]. The three-dimensional architecture is consolidated by C—H⋯O and C—H⋯π inter­actions.

## Related literature

For background to the biological activity of related phenanthrene compounds, see: Wang *et al.* (2010 ▶); Rostom *et al.* (2011 ▶). For related structures, see: Asiri *et al.* (2011 ▶); Al-Youbi *et al.* (2012 ▶). For additional conformational analysis, see: Cremer & Pople (1975 ▶).
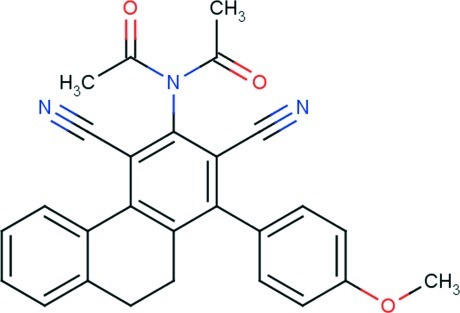


## Experimental

### 

#### Crystal data

C_27_H_21_N_3_O_3_
*M* *_r_* = 435.47
Orthorhombic, 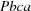

*a* = 8.3321 (4) Å
*b* = 19.4037 (11) Å
*c* = 27.5887 (14) Å
*V* = 4460.4 (4) Å^3^
*Z* = 8
Mo *K*α radiation
μ = 0.09 mm^−1^
*T* = 100 K
0.40 × 0.20 × 0.10 mm

#### Data collection

Agilent SuperNova Dual diffractometer with an Atlas detector
Absorption correction: multi-scan (*CrysAlis PRO*; Agilent, 2011 ▶) *T*_min_ = 0.966, *T*_max_ = 0.991
11528 measured reflections
5106 independent reflections
3488 reflections with *I* > 2σ(*I*)
*R*_int_ = 0.041

#### Refinement

*R*[*F* ^2^ > 2σ(*F* ^2^)] = 0.061
*wR*(*F* ^2^) = 0.166
*S* = 1.02
5106 reflections
300 parameters
H-atom parameters constrained
Δρ_max_ = 0.39 e Å^−3^
Δρ_min_ = −0.29 e Å^−3^

### 

Data collection: *CrysAlis PRO* (Agilent, 2011 ▶); cell refinement: *CrysAlis PRO*; data reduction: *CrysAlis PRO*; program(s) used to solve structure: *SHELXS97* (Sheldrick, 2008 ▶); program(s) used to refine structure: *SHELXL97* (Sheldrick, 2008 ▶); molecular graphics: *X-SEED* (Barbour, 2001 ▶) and *DIAMOND* (Brandenburg, 2006 ▶); software used to prepare material for publication: *publCIF* (Westrip, 2010 ▶).

## Supplementary Material

Crystal structure: contains datablock(s) global, I. DOI: 10.1107/S160053681201210X/lh5437sup1.cif

Structure factors: contains datablock(s) I. DOI: 10.1107/S160053681201210X/lh5437Isup2.hkl

Supplementary material file. DOI: 10.1107/S160053681201210X/lh5437Isup3.cml

Additional supplementary materials:  crystallographic information; 3D view; checkCIF report

## Figures and Tables

**Table 1:** Hydrogen-bond geometry (Å, °) *Cg*1 and *Cg*2 are the centroids of the C4–C9 and C21–C26 rings, respectively.

*D*—H⋯*A*	*D*—H	H⋯*A*	*D*⋯*A*	*D*—H⋯*A*
C5—H5⋯O2^i^	0.95	2.40	3.255 (3)	150
C25—H25⋯O2^ii^	0.95	2.59	3.154 (3)	119
C26—H26⋯O2^ii^	0.95	2.54	3.141 (3)	121
C27—H27*A*⋯O1^iii^	0.98	2.41	3.160 (3)	133
C3—H3*B*⋯*Cg*1^iv^	0.99	2.74	3.696 (3)	164
C19—H19*A*⋯*Cg*2^v^	0.98	2.82	3.618 (3)	139

Symmetry codes: (i) 
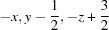
; (ii) 
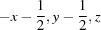
; (iii) 
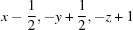
; (iv) 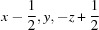; (v) 
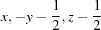
.

**Fig. 1.**
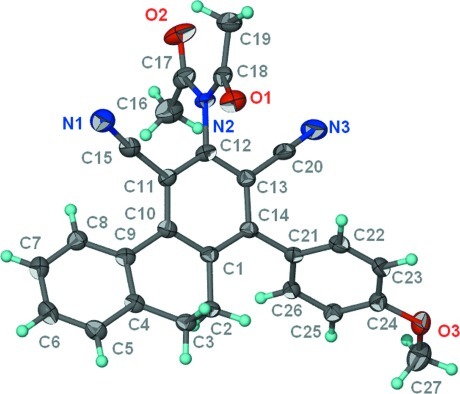
The molecular structure of (I) showing the atom-labelling scheme and displacement ellipsoids at the 70% probability level.

**Fig. 2.**
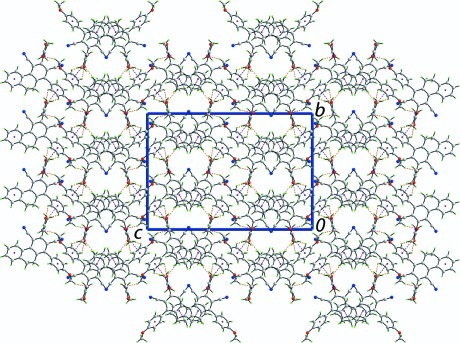
A view in projection down the a axis of the unit-cell contents of (I). The C—H···O and C—H···π interactions are shown as orange and purple dashed lines, respectively.

## supplementary crystallographic information

### Comment

The biological activity of phenanthrene compounds (Wang *et al.* 2010; Rostom *et al.*, 2011), has motivated structural studies on these systems Asiri *et al.*, 2011; Al-Youbi *et al.*, 2012) and led to the characterization of the title compound, (I).

In (I), Fig. 1, the cyclohexa-1,3-diene ring has a screw-boat conformation as quantified by the following geometric parameters (Cremer & Pople, 1975): puckering parameters q_2_ = 0.499 (2) Å and q_3_ = 0.176 (2) Å, and amplitudes: Q = 0.529 (2) Å, θ = 70.6 (2)° and φ_2_ = 91.2 (3)°. The benzene rings of the 1,2-dihydronaphthalene and methoxybenzene residues form dihedral angles of 27.61 (6) and 59.47 (7)°, respectively, with the central dicyanobenzene ring, indicating a fold in the fused ring system and a twist of the methoxybenzene ring out of the plane of the benzene ring to which it is connected. The *N*-acetylacetamide residue has an *anti* conformation and is essentially planar. The r.m.s. deviation for the seven fitted atoms = 0.0935 Å with maximum deviations of 0.1476 (15) Å for the O2 atom and -0.1378 (15) Å for the C19 atom. This residue is inclined in a perpendicular fashion with respect to the benzene ring to which it is connected, forming a dihedral angle of 89.14 (6)°.

The three-dimensional architecture of (I) is stabilized by C—H···O and C—H···π interactions, Fig. 2 and Table 1. Notably, the O2 atom of the *N*-acetylacetamide, *i.e*. the oxygen atom residue directed away from the rest of the molecule, participates in three C—H···O interactions, Table 1.

### Experimental

A mixture of 3-amino-1-(4-methoxyphenyl)-9,10- dihydrophenanthrene-2,4-dicarbonitrile (0.01 mmol), acetic anhydride (5 ml) and conc. H_2_SO_4_ (0.5 ml) was heated over a boiling water bath for 10 min. The solution was cooled, poured onto ice-cold water, treated with 20% NaOH solution till alkaline (pH = 11). The crude solid product was filtered and recrystallized from ethanol. Yield: 68%. *M*.pt: 455–457 K.

### Refinement

Carbon-bound H-atoms were placed in calculated positions [C—H = 0.95 to 0.99 Å, *U*_iso_(H) = 1.2 to 1.5*U*_eq_(C)] and were included in the refinement in the riding model approximation.

### Crystal data

**Table:**

C_27_H_21_N_3_O_3_	*F*(000) = 1824
*M**_r_* = 435.47	*D*_x_ = 1.297 Mg m^−^^3^
Orthorhombic, *P**b**c**a*	Mo *K*α radiation, λ = 0.71073 Å
Hall symbol: -P 2ac 2ab	Cell parameters from 2867 reflections
*a* = 8.3321 (4) Å	θ = 2.4–27.5°
*b* = 19.4037 (11) Å	µ = 0.09 mm^−^^1^
*c* = 27.5887 (14) Å	*T* = 100 K
*V* = 4460.4 (4) Å^3^	Prism, light-brown
*Z* = 8	0.40 × 0.20 × 0.10 mm

### Data collection

**Table:**

Agilent SuperNova Dual diffractometer with an Atlas detector	5106 independent reflections
Radiation source: SuperNova (Mo) X-ray Source	3488 reflections with *I* > 2σ(*I*)
Mirror monochromator	*R*_int_ = 0.041
Detector resolution: 10.4041 pixels mm^-1^	θ_max_ = 27.6°, θ_min_ = 2.6°
ω scan	*h* = −10→7
Absorption correction: multi-scan (*CrysAlis PRO*; Agilent, 2011)	*k* = −13→25
*T*_min_ = 0.966, *T*_max_ = 0.991	*l* = −35→21
11528 measured reflections	

### Refinement

**Table:**

Refinement on *F*^2^	Primary atom site location: structure-invariant direct methods
Least-squares matrix: full	Secondary atom site location: difference Fourier map
*R*[*F*^2^ > 2σ(*F*^2^)] = 0.061	Hydrogen site location: inferred from neighbouring sites
*wR*(*F*^2^) = 0.166	H-atom parameters constrained
*S* = 1.02	*w* = 1/[σ^2^(*F*_o_^2^) + (0.0647*P*)^2^ + 2.7824*P*] where *P* = (*F*_o_^2^ + 2*F*_c_^2^)/3
5106 reflections	(Δ/σ)_max_ = 0.001
300 parameters	Δρ_max_ = 0.39 e Å^−^^3^
0 restraints	Δρ_min_ = −0.29 e Å^−^^3^

### Fractional atomic coordinates and isotropic or equivalent isotropic displacement parameters (Å^2^)

**Table:**

	*x*	*y*	*z*	*U*_iso_*/*U*_eq_	
O1	0.2784 (2)	0.49381 (8)	0.63579 (6)	0.0326 (4)	
O2	−0.1406 (2)	0.59674 (9)	0.61677 (7)	0.0403 (5)	
O3	0.0262 (2)	0.10850 (9)	0.47080 (6)	0.0332 (4)	
N1	0.0632 (3)	0.51602 (11)	0.74941 (8)	0.0433 (6)	
N2	0.0092 (2)	0.49981 (9)	0.62945 (7)	0.0213 (4)	
N3	−0.0310 (3)	0.41448 (11)	0.51859 (8)	0.0392 (6)	
C1	0.0157 (2)	0.28935 (11)	0.67074 (8)	0.0189 (5)	
C2	0.0293 (3)	0.21508 (11)	0.68718 (8)	0.0210 (5)	
H2A	0.0725	0.1866	0.6604	0.025*	
H2B	−0.0785	0.1973	0.6956	0.025*	
C3	0.1396 (3)	0.20966 (11)	0.73113 (8)	0.0217 (5)	
H3A	0.1433	0.1613	0.7426	0.026*	
H3B	0.2497	0.2237	0.7220	0.026*	
C4	0.0780 (2)	0.25552 (11)	0.77096 (8)	0.0205 (5)	
C5	0.0778 (3)	0.23518 (12)	0.81939 (8)	0.0245 (5)	
H5	0.1225	0.1918	0.8280	0.029*	
C6	0.0133 (3)	0.27708 (13)	0.85540 (8)	0.0273 (5)	
H6	0.0167	0.2629	0.8883	0.033*	
C7	−0.0557 (3)	0.33955 (13)	0.84298 (8)	0.0269 (5)	
H7	−0.1024	0.3677	0.8674	0.032*	
C8	−0.0567 (3)	0.36109 (12)	0.79510 (8)	0.0241 (5)	
H8	−0.1048	0.4039	0.7869	0.029*	
C9	0.0125 (3)	0.32038 (11)	0.75861 (8)	0.0206 (5)	
C10	0.0137 (3)	0.34111 (11)	0.70697 (8)	0.0200 (5)	
C11	0.0169 (3)	0.41078 (11)	0.69237 (8)	0.0202 (5)	
C12	0.0090 (3)	0.42839 (11)	0.64368 (8)	0.0222 (5)	
C13	0.0038 (3)	0.37711 (11)	0.60844 (8)	0.0204 (5)	
C14	0.0111 (2)	0.30663 (11)	0.62148 (8)	0.0189 (4)	
C15	0.0407 (3)	0.46807 (12)	0.72585 (9)	0.0295 (5)	
C16	−0.2878 (3)	0.49315 (14)	0.63111 (11)	0.0379 (6)	
H16A	−0.3823	0.5230	0.6288	0.057*	
H16B	−0.2863	0.4705	0.6629	0.057*	
H16C	−0.2922	0.4582	0.6055	0.057*	
C17	−0.1384 (3)	0.53571 (12)	0.62517 (9)	0.0288 (5)	
C18	0.1648 (3)	0.52930 (12)	0.62423 (8)	0.0247 (5)	
C19	0.1828 (3)	0.60013 (12)	0.60405 (9)	0.0336 (6)	
H19A	0.2963	0.6091	0.5971	0.050*	
H19B	0.1436	0.6338	0.6277	0.050*	
H19C	0.1204	0.6040	0.5741	0.050*	
C20	−0.0140 (3)	0.39733 (12)	0.55834 (9)	0.0268 (5)	
C21	0.0139 (3)	0.25367 (11)	0.58236 (8)	0.0200 (5)	
C22	0.1360 (3)	0.25280 (12)	0.54760 (8)	0.0251 (5)	
H22	0.2192	0.2862	0.5492	0.030*	
C23	0.1380 (3)	0.20432 (12)	0.51101 (8)	0.0266 (5)	
H23	0.2224	0.2042	0.4879	0.032*	
C24	0.0150 (3)	0.15530 (12)	0.50807 (8)	0.0243 (5)	
C25	−0.1080 (3)	0.15542 (11)	0.54194 (8)	0.0223 (5)	
H25	−0.1922	0.1226	0.5399	0.027*	
C26	−0.1067 (3)	0.20427 (11)	0.57908 (8)	0.0205 (5)	
H26	−0.1900	0.2038	0.6026	0.025*	
C27	−0.1006 (3)	0.06072 (15)	0.46578 (10)	0.0420 (7)	
H27A	−0.0789	0.0304	0.4381	0.063*	
H27B	−0.2014	0.0855	0.4603	0.063*	
H27C	−0.1093	0.0331	0.4954	0.063*	

### Atomic displacement parameters (Å^2^)

**Table:**

	*U*^11^	*U*^22^	*U*^33^	*U*^12^	*U*^13^	*U*^23^
O1	0.0286 (9)	0.0278 (9)	0.0413 (10)	−0.0001 (7)	−0.0040 (8)	0.0001 (8)
O2	0.0445 (11)	0.0217 (9)	0.0548 (12)	0.0075 (8)	−0.0187 (9)	−0.0036 (8)
O3	0.0439 (10)	0.0312 (9)	0.0244 (9)	−0.0042 (8)	0.0033 (8)	−0.0079 (7)
N1	0.0689 (17)	0.0307 (12)	0.0303 (11)	−0.0073 (12)	−0.0001 (12)	−0.0032 (10)
N2	0.0276 (10)	0.0130 (9)	0.0234 (9)	0.0005 (7)	−0.0038 (8)	0.0006 (7)
N3	0.0618 (15)	0.0267 (11)	0.0291 (12)	−0.0033 (11)	−0.0083 (11)	0.0036 (9)
C1	0.0163 (10)	0.0169 (11)	0.0235 (11)	−0.0004 (8)	0.0023 (9)	0.0018 (9)
C2	0.0236 (11)	0.0179 (11)	0.0217 (11)	0.0010 (9)	−0.0001 (9)	0.0039 (9)
C3	0.0211 (11)	0.0203 (11)	0.0237 (11)	0.0020 (9)	−0.0015 (9)	0.0035 (9)
C4	0.0156 (10)	0.0226 (11)	0.0232 (11)	−0.0022 (8)	−0.0014 (9)	0.0019 (9)
C5	0.0209 (11)	0.0274 (12)	0.0252 (12)	0.0025 (9)	−0.0028 (9)	0.0064 (10)
C6	0.0261 (12)	0.0351 (14)	0.0208 (11)	−0.0021 (10)	−0.0005 (10)	0.0044 (10)
C7	0.0270 (12)	0.0300 (13)	0.0238 (11)	0.0001 (10)	0.0016 (10)	−0.0025 (10)
C8	0.0230 (11)	0.0227 (12)	0.0267 (12)	−0.0003 (9)	0.0006 (10)	−0.0011 (9)
C9	0.0177 (10)	0.0205 (11)	0.0237 (11)	−0.0018 (9)	−0.0014 (9)	0.0006 (9)
C10	0.0170 (10)	0.0199 (11)	0.0230 (11)	0.0005 (8)	−0.0003 (9)	0.0023 (9)
C11	0.0223 (11)	0.0178 (11)	0.0204 (11)	0.0010 (9)	−0.0012 (9)	−0.0010 (9)
C12	0.0238 (11)	0.0167 (11)	0.0261 (12)	0.0015 (9)	−0.0022 (9)	−0.0001 (9)
C13	0.0226 (11)	0.0190 (11)	0.0196 (10)	−0.0014 (9)	−0.0016 (9)	0.0005 (8)
C14	0.0182 (10)	0.0171 (11)	0.0215 (10)	−0.0010 (8)	0.0009 (9)	0.0015 (9)
C15	0.0393 (14)	0.0232 (12)	0.0259 (12)	−0.0004 (11)	0.0007 (11)	0.0005 (10)
C16	0.0293 (13)	0.0353 (15)	0.0490 (16)	0.0035 (11)	−0.0028 (12)	0.0001 (12)
C17	0.0349 (13)	0.0248 (13)	0.0266 (12)	0.0042 (10)	−0.0079 (11)	−0.0037 (10)
C18	0.0326 (13)	0.0219 (12)	0.0195 (11)	−0.0005 (10)	−0.0038 (10)	−0.0044 (9)
C19	0.0438 (15)	0.0241 (13)	0.0328 (13)	−0.0054 (11)	−0.0042 (12)	0.0029 (10)
C20	0.0372 (13)	0.0158 (11)	0.0273 (12)	−0.0028 (10)	−0.0025 (11)	0.0003 (9)
C21	0.0236 (11)	0.0172 (10)	0.0191 (10)	0.0040 (9)	−0.0006 (9)	0.0045 (8)
C22	0.0266 (12)	0.0220 (12)	0.0269 (12)	−0.0024 (9)	0.0037 (10)	0.0038 (9)
C23	0.0307 (12)	0.0248 (12)	0.0244 (11)	0.0008 (10)	0.0086 (10)	0.0034 (9)
C24	0.0330 (13)	0.0227 (11)	0.0173 (10)	0.0040 (10)	−0.0006 (10)	0.0000 (9)
C25	0.0223 (11)	0.0219 (11)	0.0226 (11)	−0.0016 (9)	−0.0007 (9)	−0.0004 (9)
C26	0.0220 (11)	0.0203 (11)	0.0193 (10)	0.0014 (9)	0.0017 (9)	0.0009 (9)
C27	0.0426 (16)	0.0434 (16)	0.0400 (15)	−0.0022 (13)	−0.0020 (13)	−0.0141 (13)

### Geometric parameters (Å, º)

**Table:**

O1—C18	1.213 (3)	C10—C11	1.411 (3)
O2—C17	1.207 (3)	C11—C12	1.388 (3)
O3—C24	1.375 (3)	C11—C15	1.459 (3)
O3—C27	1.412 (3)	C12—C13	1.392 (3)
N1—C15	1.150 (3)	C13—C14	1.415 (3)
N2—C17	1.418 (3)	C13—C20	1.445 (3)
N2—C18	1.425 (3)	C14—C21	1.490 (3)
N2—C12	1.440 (3)	C16—C17	1.503 (4)
N3—C20	1.155 (3)	C16—H16A	0.9800
C1—C14	1.400 (3)	C16—H16B	0.9800
C1—C10	1.417 (3)	C16—H16C	0.9800
C1—C2	1.515 (3)	C18—C19	1.490 (3)
C2—C3	1.525 (3)	C19—H19A	0.9800
C2—H2A	0.9900	C19—H19B	0.9800
C2—H2B	0.9900	C19—H19C	0.9800
C3—C4	1.504 (3)	C21—C26	1.392 (3)
C3—H3A	0.9900	C21—C22	1.399 (3)
C3—H3B	0.9900	C22—C23	1.380 (3)
C4—C5	1.393 (3)	C22—H22	0.9500
C4—C9	1.414 (3)	C23—C24	1.400 (3)
C5—C6	1.392 (3)	C23—H23	0.9500
C5—H5	0.9500	C24—C25	1.387 (3)
C6—C7	1.385 (3)	C25—C26	1.396 (3)
C6—H6	0.9500	C25—H25	0.9500
C7—C8	1.385 (3)	C26—H26	0.9500
C7—H7	0.9500	C27—H27A	0.9800
C8—C9	1.403 (3)	C27—H27B	0.9800
C8—H8	0.9500	C27—H27C	0.9800
C9—C10	1.481 (3)		
			
C24—O3—C27	117.15 (19)	C14—C13—C20	120.66 (19)
C17—N2—C18	125.69 (19)	C1—C14—C13	118.62 (19)
C17—N2—C12	119.62 (19)	C1—C14—C21	122.50 (19)
C18—N2—C12	114.52 (18)	C13—C14—C21	118.87 (19)
C14—C1—C10	120.98 (19)	N1—C15—C11	175.0 (3)
C14—C1—C2	121.35 (19)	C17—C16—H16A	109.5
C10—C1—C2	117.63 (19)	C17—C16—H16B	109.5
C1—C2—C3	110.40 (18)	H16A—C16—H16B	109.5
C1—C2—H2A	109.6	C17—C16—H16C	109.5
C3—C2—H2A	109.6	H16A—C16—H16C	109.5
C1—C2—H2B	109.6	H16B—C16—H16C	109.5
C3—C2—H2B	109.6	O2—C17—N2	120.8 (2)
H2A—C2—H2B	108.1	O2—C17—C16	123.2 (2)
C4—C3—C2	109.55 (17)	N2—C17—C16	116.1 (2)
C4—C3—H3A	109.8	O1—C18—N2	117.1 (2)
C2—C3—H3A	109.8	O1—C18—C19	122.9 (2)
C4—C3—H3B	109.8	N2—C18—C19	120.0 (2)
C2—C3—H3B	109.8	C18—C19—H19A	109.5
H3A—C3—H3B	108.2	C18—C19—H19B	109.5
C5—C4—C9	118.9 (2)	H19A—C19—H19B	109.5
C5—C4—C3	122.2 (2)	C18—C19—H19C	109.5
C9—C4—C3	118.85 (19)	H19A—C19—H19C	109.5
C6—C5—C4	121.3 (2)	H19B—C19—H19C	109.5
C6—C5—H5	119.3	N3—C20—C13	178.5 (3)
C4—C5—H5	119.3	C26—C21—C22	118.2 (2)
C7—C6—C5	119.7 (2)	C26—C21—C14	120.73 (19)
C7—C6—H6	120.2	C22—C21—C14	121.1 (2)
C5—C6—H6	120.2	C23—C22—C21	121.2 (2)
C6—C7—C8	120.2 (2)	C23—C22—H22	119.4
C6—C7—H7	119.9	C21—C22—H22	119.4
C8—C7—H7	119.9	C22—C23—C24	119.8 (2)
C7—C8—C9	120.8 (2)	C22—C23—H23	120.1
C7—C8—H8	119.6	C24—C23—H23	120.1
C9—C8—H8	119.6	O3—C24—C25	123.7 (2)
C8—C9—C4	119.1 (2)	O3—C24—C23	116.3 (2)
C8—C9—C10	122.7 (2)	C25—C24—C23	120.0 (2)
C4—C9—C10	118.10 (19)	C24—C25—C26	119.4 (2)
C11—C10—C1	118.5 (2)	C24—C25—H25	120.3
C11—C10—C9	122.35 (19)	C26—C25—H25	120.3
C1—C10—C9	119.11 (19)	C21—C26—C25	121.4 (2)
C12—C11—C10	120.8 (2)	C21—C26—H26	119.3
C12—C11—C15	115.6 (2)	C25—C26—H26	119.3
C10—C11—C15	123.5 (2)	O3—C27—H27A	109.5
C11—C12—C13	120.1 (2)	O3—C27—H27B	109.5
C11—C12—N2	120.04 (19)	H27A—C27—H27B	109.5
C13—C12—N2	119.8 (2)	O3—C27—H27C	109.5
C12—C13—C14	120.8 (2)	H27A—C27—H27C	109.5
C12—C13—C20	118.5 (2)	H27B—C27—H27C	109.5
			
C14—C1—C2—C3	141.1 (2)	C11—C12—C13—C14	−1.8 (3)
C10—C1—C2—C3	−36.6 (3)	N2—C12—C13—C14	177.00 (19)
C1—C2—C3—C4	56.6 (2)	C11—C12—C13—C20	176.4 (2)
C2—C3—C4—C5	139.1 (2)	N2—C12—C13—C20	−4.8 (3)
C2—C3—C4—C9	−38.3 (3)	C10—C1—C14—C13	−0.2 (3)
C9—C4—C5—C6	0.6 (3)	C2—C1—C14—C13	−177.90 (19)
C3—C4—C5—C6	−176.9 (2)	C10—C1—C14—C21	179.67 (19)
C4—C5—C6—C7	1.6 (3)	C2—C1—C14—C21	2.0 (3)
C5—C6—C7—C8	−1.7 (4)	C12—C13—C14—C1	3.1 (3)
C6—C7—C8—C9	−0.3 (3)	C20—C13—C14—C1	−175.0 (2)
C7—C8—C9—C4	2.5 (3)	C12—C13—C14—C21	−176.8 (2)
C7—C8—C9—C10	179.8 (2)	C20—C13—C14—C21	5.0 (3)
C5—C4—C9—C8	−2.6 (3)	C18—N2—C17—O2	−0.6 (3)
C3—C4—C9—C8	174.98 (19)	C12—N2—C17—O2	174.6 (2)
C5—C4—C9—C10	−179.99 (19)	C18—N2—C17—C16	178.7 (2)
C3—C4—C9—C10	−2.4 (3)	C12—N2—C17—C16	−6.1 (3)
C14—C1—C10—C11	−3.8 (3)	C17—N2—C18—O1	169.7 (2)
C2—C1—C10—C11	173.92 (19)	C12—N2—C18—O1	−5.6 (3)
C14—C1—C10—C9	177.67 (19)	C17—N2—C18—C19	−11.9 (3)
C2—C1—C10—C9	−4.6 (3)	C12—N2—C18—C19	172.7 (2)
C8—C9—C10—C11	29.9 (3)	C1—C14—C21—C26	60.5 (3)
C4—C9—C10—C11	−152.8 (2)	C13—C14—C21—C26	−119.5 (2)
C8—C9—C10—C1	−151.6 (2)	C1—C14—C21—C22	−120.4 (2)
C4—C9—C10—C1	25.7 (3)	C13—C14—C21—C22	59.5 (3)
C1—C10—C11—C12	5.2 (3)	C26—C21—C22—C23	−0.2 (3)
C9—C10—C11—C12	−176.4 (2)	C14—C21—C22—C23	−179.3 (2)
C1—C10—C11—C15	−170.0 (2)	C21—C22—C23—C24	0.6 (4)
C9—C10—C11—C15	8.5 (3)	C27—O3—C24—C25	3.7 (3)
C10—C11—C12—C13	−2.4 (3)	C27—O3—C24—C23	−177.0 (2)
C15—C11—C12—C13	173.1 (2)	C22—C23—C24—O3	−179.5 (2)
C10—C11—C12—N2	178.78 (19)	C22—C23—C24—C25	−0.2 (3)
C15—C11—C12—N2	−5.7 (3)	O3—C24—C25—C26	178.6 (2)
C17—N2—C12—C11	−89.7 (3)	C23—C24—C25—C26	−0.7 (3)
C18—N2—C12—C11	86.0 (2)	C22—C21—C26—C25	−0.7 (3)
C17—N2—C12—C13	91.5 (3)	C14—C21—C26—C25	178.4 (2)
C18—N2—C12—C13	−92.8 (2)	C24—C25—C26—C21	1.1 (3)

### Hydrogen-bond geometry (Å, º)

Cg1 and Cg2 are the centroids of the C4–C9 and C21–C26 rings, respectively.

**Table:**

*D*—H···*A*	*D*—H	H···*A*	*D*···*A*	*D*—H···*A*
C5—H5···O2^i^	0.95	2.40	3.255 (3)	150
C25—H25···O2^ii^	0.95	2.59	3.154 (3)	119
C26—H26···O2^ii^	0.95	2.54	3.141 (3)	121
C27—H27*A*···O1^iii^	0.98	2.41	3.160 (3)	133
C3—H3*B*···*Cg*1^iv^	0.99	2.74	3.696 (3)	164
C19—H19*A*···*Cg*2^v^	0.98	2.82	3.618 (3)	139

Symmetry codes: (i) −*x*, *y*−1/2, −*z*+3/2; (ii) −*x*−1/2, *y*−1/2, *z*; (iii) *x*−1/2, −*y*+1/2, −*z*+1; (iv) *x*−1/2, *y*, −*z*+1/2; (v) *x*, −*y*−1/2, *z*−1/2.
